# Responsiveness to EMT-performed basic CPR and its duration predict unachievable sustained return of spontaneous circulation and unavoidable hospital death in unwitnessed out-of-hospital cardiac arrests without bystander CPR

**DOI:** 10.1186/cc9713

**Published:** 2011-03-11

**Authors:** H Inaba, Y Takei, M Enami, Y Goto, K Ohta

**Affiliations:** 1 Kanazawa University Graduate School of Medicine, Kanazawa, Japan

## Introduction

Various criteria to terminate resuscitation have been reported. EMTs in Japan are not permitted to terminate resuscitation in the field. The aim of this study is to test the hypothesis that ECG rhythm response to basic CPR and its duration may predict hospital death.

## Methods

**Figure 1:**
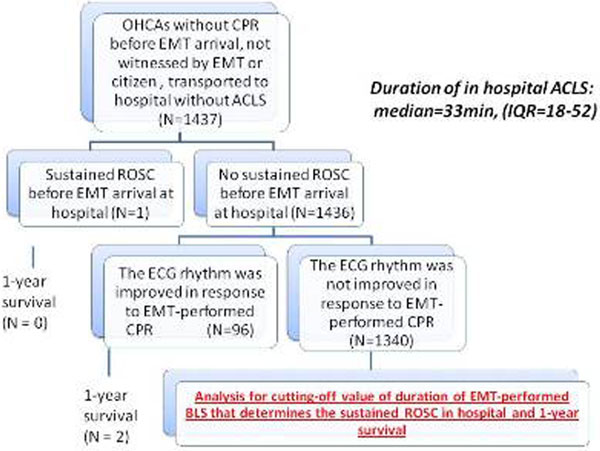
**Overview of out-of-hospital cardiac arrests analyzed**.

The basal data were prospectively collected from 1,437 unwitnessed out-of-hospital cardiac arrests (OHCAs) that were resuscitated by EMTs without the ACLS technique in Ishikawa Prefecture (Figure 1). The cut-off points of basic CPR duration for outcomes were determined. Sensitivity and specificity were calculated.

## Results

**Figure 2:**
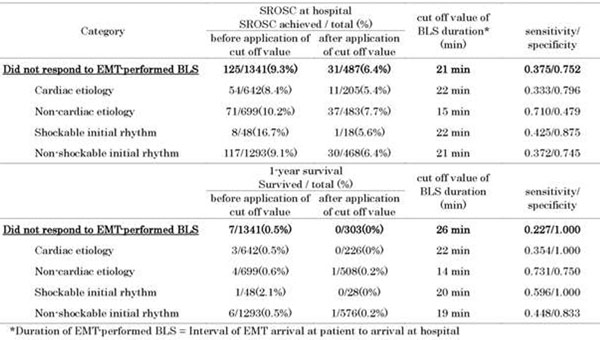
**Duration of EMT-performed BLS determines the incidence of SROSC at hospital and 1-year survival in unwitnessed OHCAs without CPR before EMT arrival**.

The improvement of the ECG rhythm by basic CPR predicted the sustained return of spontaneous circulation (SROSC) in hospital. The duration of EMT-performed CPR predicted the outcomes of the OHCAs that were unresponsive to the basic CPR (Figure 2).

## Conclusions

Responsiveness to basic CPR and its duration may predict unavoidable death in hospital.
